# Air pollution as a risk factor in health impact assessments of a travel mode shift towards cycling

**DOI:** 10.1080/16549716.2018.1429081

**Published:** 2018-02-05

**Authors:** Wasif Raza, Bertil Forsberg, Christer Johansson, Johan Nilsson Sommar

**Affiliations:** ^a^ Division of Occupational and Environmental Medicine, Department of Public Health and Clinical Medicine, Umeå University, Umeå, Sweden; ^b^ Department of Environmental Science and Analytical Chemistry, Stockholm University, Stockholm, Sweden; ^c^ Environment and Health Administration, SLB, Stockholm, Sweden

**Keywords:** Active commuting, mode shift, emission factors, population exposure, commuters’ exposure, exposure response function, comparative risk assessment

## Abstract

**Background**: Promotion of active commuting provides substantial health and environmental benefits by influencing air pollution, physical activity, accidents, and noise. However, studies evaluating intervention and policies on a mode shift from motorized transport to cycling have estimated health impacts with varying validity and precision.

**Objective**: To review and discuss the estimation of air pollution exposure and its impacts in health impact assessment studies of a shift in transport from cars to bicycles in order to guide future assessments.

**Methods**: A systematic database search of PubMed was done primarily for articles published from January 2000 to May 2016 according to PRISMA guidelines.

**Results**: We identified 18 studies of health impact assessment of change in transport mode. Most studies investigated future hypothetical scenarios of increased cycling. The impact on the general population was estimated using a comparative risk assessment approach in the majority of these studies, whereas some used previously published cost estimates. Air pollution exposure during cycling was estimated based on the ventilation rate, the pollutant concentration, and the trip duration. Most studies employed exposure-response functions from studies comparing background levels of fine particles between cities to estimate the health impacts of local traffic emissions. The effect of air pollution associated with increased cycling contributed small health benefits for the general population, and also only slightly increased risks associated with fine particle exposure among those who shifted to cycling. However, studies calculating health impacts based on exposure-response functions for ozone, black carbon or nitrogen oxides found larger effects attributed to changes in air pollution exposure.

**Conclusion**: A large discrepancy between studies was observed due to different health impact assessment approaches, different assumptions for calculation of inhaled dose and different selection of dose-response functions. This kind of assessments would improve from more holistic approaches using more specific exposure-response functions.

## Background

Urbanization has increased greatly since the end of the nineteenth century [1]. Currently, 73% of the European population is living in cities and towns, and this proportion is expected to increase to 82% by 2050 [2]. This rapid urbanization has had significant impacts on the modernization of transportation as seen by increasing use of automobiles since the Second World War [3].

Transportation’s role in economic and social development is quite clear, but it also has major health impacts. Air pollution, physical activity, road traffic injuries, and noise are important determinants of health that are affected by transportation patterns [4]. Motorized transport increases physical inactivity and a sedentary lifestyle. Physical inactivity has been identified as one of the major risks for both mortality and morbidity [5], and it is globally one of the major risk factors for mortality after illnesses such as coronary heart disease, diabetes type 2, stroke, breast cancer, colon cancer, and cognitive decline [5–8]. Transportation is also responsible for 23% of global greenhouse gas emissions [9], although the transport sector has reduced its emissions of air pollutants in Europe considerably since 2000. In 2014, transport contributed 15% of the PM2.5 from primary combustion emissions through vehicle exhaust. Non-exhaust traffic emissions that contribute to total road traffic emission, are estimated to equal about 22% of the exhaust emissions of primary PM2.5, but there are large variations across Europe [10]. Transport-related air pollution increases not only mortality, but also the risk of different health outcomes such as cardiovascular and respiratory diseases, cancer, and adverse birth outcomes [11], and ambient PM2.5 accounts for about 4.2 million deaths and 103.1 million disability-adjusted life-years (DALYs) in 2015 [12]. Lack of physical activity, high BMI, and ambient particulate matter pollution were included among the leading risk factors of the global burden of disease in 2015 [13]. Increased motorization is also an important cause of increased road traffic injuries, which are ranked as the third highest cause of mortality and are projected to account for 5% of the global burden of disease by the year 2030 [14].

Change in travel behavior in terms of promotion of active transportation (cycling and walking to work or to daily destinations) is among the strategies that have been implemented in some European countries in order to reduce the emissions from motor vehicles [15]. Additionally, active transport promotion is one way to incorporate greater physical activity in daily life [16–18], and sufficient evidence is available to establish the importance of transport interventions in the promotion of physical activity [19]. Further, different strategies that promote both public and active transportation might decrease the risk of traffic injuries [20]. Although the literature indicates negative effects of active transportation in terms of increased air pollution exposure and risk of road traffic injuries for active commuters compared with those travelling by car, evidence shows that for the commuters the health benefits of cycling outweigh the risks, especially in areas where the air pollution level is comparatively lower and where active transportation facilities, such as bicycle pathways, are properly maintained [21,22]. Using PM2.5 as an indicator of health effects, it is only for the most extreme air pollution situations that the harm caused by air pollution will outweigh the benefits for those choosing active travel [23]. However, the break-even point will depend on the air pollutant used as the health indicator as well as the exposure-response function that is used.

Health benefits from a shift towards more active transport will depend on the degree of substitution of other types of physical activities. Recent evidence from longitudinal studies suggest that bicycle commuting on average adds to overall physical activity without affecting the participation in other types of activities [24–26].

Different policies and transport interventions focusing on changes in mode of transport from cars to bicycles mainly intend to evaluate the following four health aspects: (1) Health benefits of increased physical activity for commuters, (2) Health benefits for the general population due to decreased air pollution, (3) The impacts of changes in air pollution exposure among commuters, and (4) The impacts on accident risks for commuters. Exploration of these health effects is quite helpful for transport planning purposes. The health impact assessment (HIA) approach was used in various ways as a methodology in these studies for the evaluation of transport intervention or policies’ impacts on health. These HIAs focus on the health impacts of different transport interventions in order to compare harms and health benefits [27].

The studies differ regarding to what extent the above-mentioned health aspects have been considered. To date, very little work has been done in order to understand the different aspects of moving from motorized transport to bicycling. Even though different reviewers have investigated the health and economic benefits of active transportation [28–30], to the best of our knowledge there are only three systematic reviews investigating the health impacts of active transportation, one focusing on health risks and benefits [31] and two focusing on methodological issues [30,32]. Each of the aspects, or health pathways, listed above are important to consider in such HIAs, but these are quite complicated and can be dealt with in more or less sophisticated ways.

### Aims

In conducting a systematic review of HIA studies on the change in mode of transport towards increased bicycling, we aimed to explore how the effects of air pollution have been dealt with. In particular, we aimed to review (i) how air pollution exposure was estimated for the travelers, (ii) how other impacted parts of the population were identified and how their exposures were estimated, (iii) which air pollutants were chosen as the most relevant, and (iv) what kinds of exposure response relationships were used. In addition, we discuss the appropriateness of the applied methods and assumptions in order to guide further HIAs.

## Methods

We conducted this review according to the Preferred Reporting Items for Systematic Reviews and Meta-Analyses (PRISMA) guidelines to make sure that it is comprehensive and repeatable [33]. PubMed was primarily used for searching relevant studies using the following combination of keywords; ‘health impact assessment AND transport intervention OR active transportation OR alternative transport OR cycling OR physical activity OR air pollution OR air quality OR transport traffic emission’. In addition, we also retrieved relevant studies from the Google Scholar and reference lists of the identified studies.

After removing duplicates, we read the abstracts of all studies and excluded those that did not fulfill our inclusion criteria. A full article review was conducted among the remaining studies that met the following inclusion criteria:

1. Published from January 2000 to May 2016.
2. Aiming at interventions in the transport sector to enhance physical activity and/or reduce the amount of short car trips in urban areas.
3. Evaluating the health benefits of alternative transport scenarios.
4. Quantifying air pollution exposure and presenting results about the effects of vehicle emissions on health due to mode shift from motorized transport to active commuting and public transport.
5. Using comparative risk assessment, cost benefit analysis, risk assessment, or benefit assessment as the methodology for health impact assessment.
6. Quantifying the effects on at least one health outcome.
7. Reporting either health measures such as health risk or benefit or equivalent economic measures such as economic cost and benefit relationships.

## Results

**Figure 1.**
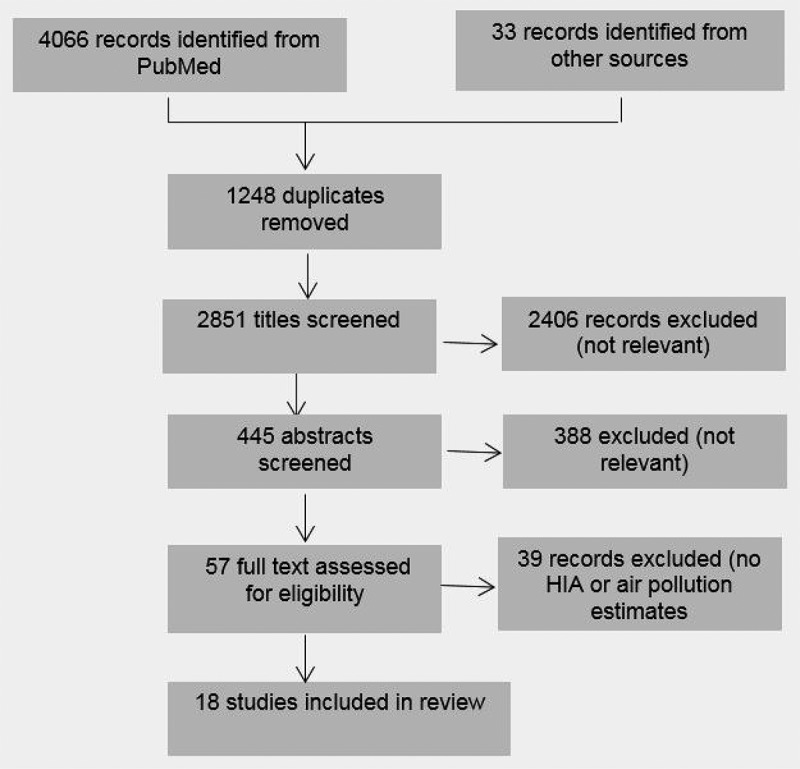
Flowchart of studies included in the review.

Our literature review identified a total of 2,851 articles. After screening the titles and abstracts, we identified 56 papers, of which only 17 studies met all of the inclusion criteria (Figure 1). The selected studies were published from 2009 through 2016, and among these 10 estimated the impacts for a scenario within Europe, one within Australia, two each within the US and New Zealand, and one in both Asian and European settings. In addition, we included one study under review from year 2016 that was published in January 2017. All included papers focused on increased amount of cycling and the effects on air pollution, greenhouse gas emissions, and health benefits due to increased physical activity. Most of these studies investigated future hypothetical transport scenarios based on information on changes in the volume of motorized traffic, e.g. the number of trips by bicycle, travel distance, or travel time.

Most papers present information on the relative change in active transport or car trips. The magnitude in change ranges from 5 to 50 percent increase in bike trips. Most commonly the sub-population of society undergoing a mode change from car to active travel or public transport was examined in these studies. However, there were also some exceptions. For example, Maizlish et al. [34] and Woodcock et al. [35,36], who evaluated the mode of transport shift to active travel in whole population. In one study, Johansson et al. [37], individual data on people’s home and work addresses were used, as well as their age, sex, and expected physical capacity, in order to establish realistic bicycle travel distances. Rojas-Rueda et al. [38] and Woodcock et al. [39] evaluated the current benefit of increased cycling due to a public bicycle-sharing program. Dhondt et al. [40] simulated the travel activity patterns before and after an increase in fuel prices, while Macmillan et al. [41] simulated the effects of policy changes employing the System Dynamics Modelling.

Thirteen studies used a comparative risk assessment approach for quantification of health risks or benefits of a change from motorized transport to cycling [22,34–40,42–46]. The economic impacts of transport interventions were estimated in four studies using a cost benefit analysis approach [41,47–49], while a benefit analysis without focusing on risks or costs was conducted in one study [50]. Important health endpoints that were evaluated in the studies were (a) mortality, including cause-specific mortality, all-cause mortality, and fatalities; (b) morbidity, including cardiovascular diseases, respiratory diseases, diabetes type 2, cancer, dementia, depression, and injuries; and (c) burden of disease (BoD), including both morbidity and mortality in the form of years of life lost (YLL) and years lost due to disability (YLD). Some studies also included individual health events such as disease-specific hospital admission, activity restriction days, and emergency room visits [40,48,50]. Buekers et al. [47] and Rabl et al. [49] expressed the impacts using the equivalent monetary benefits approach of Value of a Life Year (VOLY) [51]. Value of a Statistical Life (VSL) [52] used in the Health Economic Assessment Tool (HEAT), developed by the World Health Organization (WHO) [53], is another approach employed in two studies to quantify the economic consequences of the health benefits of walking or cycling [48,49]. Both of these approaches are based on the willingness to pay for the benefits related to a decrease in mortality risks [51,52].

The health impacts of cycling due to a mode shift were estimated primarily by changes in health impacts associated with changes in air pollution, physical activity, and accidents. However, Rabl et al. [49] also considered the health impacts of noise exposure to the general population. The health impacts of increased physical activity resulting from increased cycling were evaluated in all studies. Physical activity was mainly reported as distance or time spent in active travel, except in seven studies – Dhondt et al. [40], Maizlish et al. [34], Woodcock et al. [35,36,39], Buekers et al. [47] and Rojas-Rueda et al. [43] – where it was expressed as metabolic equivalent of task (MET) hours per week. All except two studies – Holm et al. [42] and Xia et al. [46] – measured physical activity as a continuous variable and employed published risk estimates to evaluate the health impacts of increased cycling. The HEAT tool was applied in seven studies to assess impact of physical activity on all-cause mortality [36,38,43,44,47,48,50]. Holm et al. [42] and Xia et al. [46] used categories of physical activity and the corresponding relative risks (RRs).

### Air pollution

An overview regarding air pollution information in the 18 included studies is given in Tables 1 and 2. Nine studies examined the air pollution impact on the general population [34–37,40,41,46,48,50], four estimated impacts on active commuters [38,39,42,43], and five included impacts on both the general population and active commuters [22,44,45,47,49]. The included studies modeled both particulate and gaseous pollutants such as particulate matter (PM) less than 2.5 µm and less than 10 µm (PM2.5 and PM10), sulfur dioxide, nitrogen dioxide (NO_2)_ and nitrogen oxides (NOx), carbon monoxide, ammonia, etc. However, PM2.5 was primarily used for health impact calculations because of its strong association with health outcomes [54], because it is an indicator that can avoid double counting [42], and because of the difficulty in determining the exposure with other gaseous pollutants and the nonsignificant association between gaseous pollutants and health outcomes [49,55]. Woodcock et al. [35] modeled PM10 assuming that changes in PM10 concentration within transport scenarios were in the PM2.5 size range. Dhondt et al. [40] selected elemental carbon (EC) for their analysis due to its sensitivity to combustion-related emissions, for example, diesel exhaust, and to its high relevance to health compared with PM. Johansson et al. [37] used NOx, NO_2_ and black carbon (BC) as exhaust indicators and compared the size of the estimated health impacts. Some studies also employed air pollutants in their sensitivity analysis that were different from the pollutants in their main analysis [38,44].

**Table 1.** Air pollution impact calculation methods for the general population among, (a) the studies that only calculated air pollution effects among general population and (b) the studies that calculated air pollution effects among both general population and active commuters.

Author (year)	Exposure Assessment	Pollutants	Ageᴬ	Exposure-response unction/Cost estimates
(a)				
Woodcock et al. 2009 [35]	EM and DM Emission factors*	PM2.5	All ages	Mortality: lung cancer and CR [104], ARI <5 years Ostro et al. [56].
Lindsay et al. 2010 [48]	EM and DM Emission factors*	GHGE	Adultsᴮ	HAPINZᴱ [82].
Grabow et al. 2012 [50]	EM and DM Emission factors*	PM2.5 and O_3_	All ages	BenMAP^C^ [57].
Dhondt et al. 2013 [40]	EM and DM Emission factors*	EC	Adultsᴮ	All-cause mortality: Janssen et al. [102] CVD admission: Tolbert et al. ^F^ [58].
Maizlish et al. 2013 [34]	EM and DM Emission factors*	PM2.5	All ages^D^	Mortality: CP [106], lung cancer [104], and RI [59].
Woodcock et al. 2013 [36]	Published estimates and Emission factors**	PM2.5	All ages	Mortality: lung cancer and CR [104], ARI < 5 years [56].
Macmillan et al. 2014 [41]	EM Emission factors*	PM10 and CO	Adultsᴮ	HAPINZᴱ [82].
Xia et al. 2015 [46]	EM with emission factors**	PM2.5	All ages	RR from the literatureᴳ
Johansson et al. 2017 [37]	Emission factors* and DM	NOx, NO_2_, and BC	20–65	Mortality: NOx: Nafstad et al. [103], NO2: Faustini et al. [60] BC: Hoek et al. [99].
(b)				
De Hartog et al. 2010^H^ [22]	DM	NO_2_	Adults^D^	All-cause mortality [61].
Rabl et al. 2012 [49]	EM with emission factors**	PM2.5	All ages^D^	ExternE^I^ [81] based on all – cause mortality [104]. VOLY^K^ [51].
Rojas-Rueda et al. 2012^H^ [44]	DM Used the ratio of (PM2.5/PM10)	PM2.5	All ages	All-cause mortality [106].
Rojas-Rueda et al. 2013^H^ [45]	DM Used the ratio of (PM2.5/PM10)	PM2.5	All ages^D^	RR for morbidity from the literature^J^
Buekers et al. 2015 [47]	Proportion of transport emission	PM 2.5	All ages^D^	All-cause mortality [62] and VOLY^K^ [51]

EM: emission model (for initial prediction of traffic emission); DM: (dispersion model to calculate change in pollutants concentration); GHGE: greenhouse gas emission; EC: elemental carbon; PM2.5: Particulate matter less than 2.5 µm; PM10: particulate matter less than 10 µm; CO: carbon monoxide;  O_3_: ozone; NO_2_: nitrogen dioxide; CVD: cardiovascular disease, CP: cardiopulmonary; CR: cardiorespiratory; ARI: acute respiratory infection; RI: respiratory infection; RM: respiratory mortality. *Published emission factors but not reported in text. **Emission factors reported explicitly in text ᴬ Age groups according to health outcomes; ᴯ Such as ≥18 years or ≥30 years; ^C^ Environmental Benefits Mapping and Analysis Program using the concentration response function from chronic bronchitis [63], acute bronchitis [64], all-cause mortality [65,104], COPD hospitalization (Moolgavgkar 2000a, 2003) [66], asthma emergency room visits [67], work loss days [68], asthma (symptoms) [69], minor-restricted activity days [70], acute MI [71], respiratory disease [72], lower respiratory symptoms [73], and cough among asthmatic children [74]; ^D^ Probable, but not specified explicitly in the text; ᴱ Health And Air Pollution Study in New Zealand to estimate the morbidity and mortality health costs associated with traffic emissions [82]; ^F^CVD admission >64 years: [75]; ᴳ Mortality: <75 and >75 years, respiratory disease (65 years) [76], and lung cancer [104] Morbidity: CVD, respiratory disease [76], and lung cancer [104]; ^H^ Method of transport emission estimation is quite vague in determination of emission factors; ^I^ External cost of energy to estimate the automotive pollution impact on health in Europe [81]; ^J^ Cerebrovascular disease and lower respiratory tract infection [77], preterm weight [78], low term weight [79], and CVD (Mustafic 2012) [80]; ^K^ Value of a Life Year: calculation of monetary benefits of mortality reduction using a life tables approach.

**Table 2.** Air pollution impact calculation methods for commuters among, (a) the studies that only calculated air pollution effects among active commuters and (b) the studies that calculated air pollution effects among both general population and active commuters.

Author (year)	Actual/Estimated	Age (years)ᴬ	Exposure Assessment	Pollutants	Dose	Exposure response function
(a)						
Rojas- Rueda et al. 2011 [38]	Actual^E^	16–64	Mode- specific concentration	PM2.5	Mode- specific concentration, inhalation rate, and trip duration.	All-cause mortality: Krewski et al. [106] and Beelen et al. [107].
Holm et al. 2012 [42]	Estimated^F^	Adults^C^	Average from two street monitoring sites	PM2.5	Average from two street monitoring sites, inhalation rate, and trip duration	Mortality: CP and lung cancer [104]
Woodcock et al. 2014 [39]	Actual^E^	≥15	Based on mode-specific scaling factors	PM2.5	Mode-specific concentration, inhalation rate, and trip duration	Mortality: CP and lung cancer (>30 years): Pope et al. [104], RM (<5 years) Ostro et al. [56].
Rojas-Rueda et al. 2016 [43]	Estimated^F^	16–64	Mode- specific concentration	PM2.5	Mode- specific concentration, inhalation rate, and trip duration	All-cause mortality [99].
(b)						
De Hartog et al. 2010 [22]	Estimated^F^	Adultsᴯ	Mode- specific concentration	PM2.5, BS	Mode specific concentration, inhalation rate, and trip duration	All-cause mortality:PM2.5 [104], BS: [107].
Rabl et al. 2012 [49]	Estimated^F^	20–65	Based on mode- specific scaling factors	PM2.5	Mode- specific concentration, inhalation rate, and trip duration	ExternE^D^ [81] based on all-cause mortality [104]. VOLY^H^ [51].
Rojas-Rueda et al. 2012 [44]	Estimated^F^	16–64	Mode- specific concentration	PM2.5 from PM10	Mode- specific concentration, inhalation rate, and trip duration	All-cause mortality [106].
Rojas-Rueda et al. 2013 [45]	Estimated^F^	16–64	Mode- specific concentration	PM2.5	Mode- specific concentration, inhalation rate, and trip duration	RR for morbidity from the literature^G^
Buekers et al. 2015 [47]	Actual^E^	Adults^C^	Mode specific concentration	PM2.5	Mode- specific concentration, inhalation rate, and trip duration	All-cause mortality (WHO; 2013) [97] and VOLY^H^ [51].

PM2.5: Particulate matter less than 2.5 µm; PM10: particulate matter less than 10 µm; BS: black soot; CP: cardiopulmonary; RM: respiratory mortality. ᴬ Age groups according to health outcomes; ᴯ Such as ≥18 years or ≥30 years; ^C^ Probable, but not specified explicitly in the text; ^D^ External cost of energy to estimate the automotive pollution impact on health in Europe [81]; ^E^ Calculation based on the actual number of participants who changed mode from car to bicycle; ^F^ Estimated for hypothetical individuals who changed transport mode from car to bicycle; ^G^ Cerebrovascular disease and lower respiratory tract infection [77], preterm weight [78], low term weight [79], and CVD (Mustafic 2012) [80]; ^H^ Value of a Life Year: calculation of monetary benefits of mortality reduction using a life tables approach.

### Emissions

Almost all studies used a similar general approach for the initial estimation of reduced emissions resulting from decreased motorized transport and affecting the general population, but they used different emission-predicting models such as Computer Program to Calculate Emissions from Road Transport (COPERT 4), Emission Factor Model (EMFAC), Auckland’s Vehicle Emission Prediction Model (VEPM), and Handbook Emission Factors for Road Transport (HBEFA). One difference between the approaches is whether or not established emission factors were used and how detailed these are reported in the paper. Some papers give a reference to how emissions were calculated (typically from vehicle fleet, flow, speed, and published emission factors) [34,35,37,40,41,48,50], whereas a few report the emission factors explicitly in the text [36,46,49]. Buekers et al. [47] did not use emission factors; instead, they used the proportion of the concentration originating from traffic. Some studies were ambiguous in the description of emission factors [22,44,45].

### Population exposure and impact on the general population

For further estimation of air pollution impacts, the studies can be divided on the basis of whether the impacts were estimated directly with an exposure-based approach (EA) or indirectly with an emission cost-based approach (ECA). ECA was primarily used in studies aiming at avoiding health costs of transport emissions, whereas EA was employed in studies focusing on comparative risk assessment to compare health benefits and risks of changed exposure distribution due to a mode change from motorized to active transport. In case of EA, almost all studies used various air pollution dispersion models, e.g. the Barcelona Air Dispersion Model, The Air Pollution Model, etc., for the estimation of differences in pollutant concentration due to changes in transport mode. However, Dhondt et al. [40] estimated the population exposure according to a ‘dynamic exposure’ based on information regarding population location and their transport behavior, where the overall exposure was calculated by taking the average of all persons living in certain zones.

The studies assessed associated risk by using RRs and exposure-response functions given in previously published literature to calculate the impact of changes in transport emissions either on mortality [22,37,43,44] or on BoD. Among the studies including morbidity and mortality outcomes that quantified BoD, the Disability adjusted Life Years (DALYs) was estimated by calculating YLD and YLL [34–36,39,40,42,45,46]. Some studies also performed sensitivity analyses to tackle uncertainties associated with the use of published RRs. Dhondt et al. [40], Grabow et al. [50], and Rojas-Rueda et al. [38,44] used Monte Carlo simulations to check the variation in RRs, whereas Holm et al. [42] used the upper and lower limits of the confidence interval to obtain bounds for the disease burden. Johansson et al. [37] compared the differences in impacts using different exposure indicators. Some studies conducted more than one sensitivity analysis in order to check the robustness of results. For example, Rojas-Rueda et al. [43] performed two sensitivity analyses, one employing a different exposure response function and other assuming a higher toxicity of pollutant whereas Buekers et al. [47] repeated sensitivity analysis with a variable number of cyclists and travel distances.

In order to estimate the impacts of reduced emissions on the general population in case of ECA, previously published cost estimates were used without consideration of comparative risk assessments. For example, Rabl et al. [49] used estimates from ExternE (External Cost of Energy) [81], which are mortality-related health costs from transport emissions for different European cities, and thereafter, monetary health benefits were calculated by the application of VOLY [51]. Lindsay et al. [48] and Macmillan et al. [41] used estimates of the Health and Air Pollution Study in New Zealand (HAPiNZ) study [82], which measured the morbidity and mortality-related health costs of transport emission within New Zealand.

Grabow et al. [50] first used the Community Multiscale Air Quality Model to depict the change in ambient air PM2.5 and O_3_ concentration and then used the Environmental Benefits Mapping and Analysis Program (BenMap) to estimate the monetary health benefits without presenting cost or risks. Buekers et al. [47] estimated the avoided health cost by VOLY [51] by considering the impact of changes in PM2.5 on all-cause mortality.

### Commuters´ exposure and impacts of active transport

What we know about commuters’ exposure is largely based on measurement studies that aim to compare air pollution exposure and inhalation doses between different transport modes and HIA is not their main objective [83]. A review of those studies is out of the scope of this paper.

All nine HIA studies in our review that estimated the active commuters’ exposure to air pollution did this on the basis of the pollutant concentration in the ambient air, the trip duration, and the ventilation rate during cycling [22,38,39,42–45,47,49]. The basic differences between studies are in the way they calculated pollutant concentrations in traffic and what ventilation rate they assumed. The HIAs in Barcelona [38,43–45] all used PM2.5 or black smoke (BS) concentration estimates obtained from previous studies [84,85].

Four different approaches were employed to estimate commuters’ exposure. The first approach considers the mode-specific concentration in relation to background concentration [38,43,47]. The pollutant concentration for each transport mode were employed from studies conducted in Barcelona [85,86] and adjusted by the background’s annual mean concentration [38,43] except for Buekers et al. [47], that used mode-specific concentration from a meta -analysis on multiple cities [87]. This approach was likely used also in two other studies from Barcelona [44,45]. In a second approach used by a study in the Netherlands, a mean ratio comparing car and cycling-specific concentrations was used without considering the background concentration [22]. These estimates were based on the measurements of the actual mode-specific exposure on different routes. The third approach adopted by one study [49] was the use of concentrations measured in the streets of major European cities, and thereafter mode-specific exposures were estimated by assuming different scaling factors based on data reported by measuring stations of European Environmental Agency (EEA) [88]. The fourth approach taken by Woodcock et al. [39] was to model the most likely mode-specific routes and then estimate the average pollutant concentration along these routes by applying average daily estimates of PM2.5 concentrations over a fine grid for central London in 2008. Thereafter, mode-specific exposure estimates were calculated by multiplying average pollutant concentration with mode-specific scaling factors related to vehicle type and road position [39].

Holm et al. [42] used the average value from two street-monitoring sites as commuters’ exposure and ignored mode-specific exposure. Of the nine studies that evaluated active commuter exposure, all except Holm et al. [42] took into consideration the difference in the air pollution concentration between car users and cyclists. David Rojas-Rueda et al. [38,44,45] assumed that PM 2.5 concentrations in cars were 57% higher than for cyclists based on a study conducted in Barcelona [86]. The corresponding PM2.5 concentration in a study by De Hartog et al. [22] was 16% higher in cars, whereas EC concentrations were on average 65% higher among car users than cyclists based on studies from London [89–91], Arnhem [92], and several Dutch cities [93]. Rabl et al. [49] assumed 50% higher PM2.5 concentrations among car users than cyclists based on EEA measurements [88]. Woodcock et al. [39] assumed 30% higher PM2.5 concentrations in car users compared with cyclists on the basis of differences in vehicle characteristics and their position on the road. All of these studies differ from each other regarding different study settings and their way of estimating pollutant concentrations. PM2.5 concentrations in Barcelona were, for instance, measured in cars with open windows, thereby resulting in a greater difference between car user and bicyclist ambient air concentrations compared to other studies [86].

To calculate the inhaled dose among commuters, different values of ventilation rates were used in these studies. Studies assuming the ventilation rate equivalent to the mode-specific average MET [38,43–45,47] reported the ventilation rate to be between 2 and 8 times higher for cyclists compared to car users assuming a non-linear relation between energy expenditure and ventilation rate [94]. For example, Rojas-Rueda et al. [43] assigned MET values of 6.58 for cyclists and 3.55 for car user whereas the corresponding values used by others were 6 and 2 [38,47] or 6 and 1 [44]. Rabl et al. [49] and Woodcock et al. [39] assumed respectively 4 and 4.5 times higher ventilation rate for cyclists. Some studies [22,42] used a 2.2 times higher ventilation rate for cyclists compared to car users, which was derived from two Dutch studies by van Wijnen et al. [95] and Zuurbier et al. [96]. Discrepancies in observed and assumed ventilation rates may partly be explained by the differences in urban design and type of trips made by bike in different settings.

Based on these travel mode-specific exposures, the resulting change in yearly mean exposure could be calculated. Using these calculations, the long-term impacts on mortality and BoD were calculated based on published exposure-response functions. Two studies, Buekers et al. [47] and Rabl et al. [49], also estimated equivalent monetary values by applying ExternE [81] and VOLY [51] estimates in addition to calculating the estimated health impacts.

### Adopted exposure-response functions for impact calculation

For the exposure-response function between traffic air pollution and mortality, most of the studies used a RR of 1.06 per 10 µg/m^3^ increment in the annual average PM2.5, except Woodcock et al. [35,36,39] and Dhondt et al. [40], regardless of the exposure levels. This is in line with the recent WHO Review of Evidence on Health Aspects of Air Pollution (REVIHAAP) [97] that concluded that recent long-term studies show associations between PM and mortality at levels well below the current WHO air quality guideline level for PM2.5 (10 μg/m^3^). The WHO expert panel thus concluded that for Europe it is reasonable to use linear exposure-response functions, at least for particles and all-cause mortality, and to assume that any reduction in exposure will have at least some benefits. The REVIHAAP report also concludes that more studies have now been published showing associations between long-term exposure to NO_2_ and mortality [97]. This observation makes the situation a bit more complicated when it comes to impact assessments for vehicle exhaust exposure.

In the WHO Health Risk of Air Pollution in Europe (HRAPIE) impact assessment report [98], it was, for long-term exposure to PM2.5 and all-cause (natural) mortality for ages 30+, recommended to use the exposure-response function from a meta-analysis of 13 cohort studies [99]. The RR for PM2.5 from this meta-analysis was 1.062 (95% CI 1.040–1.083) per 10 μg/m^3^. This is a coefficient very close to that in the HIAs that assumed long-term effects on mortality of PM2.5 from the American Cancer Society (ACS) Cohort Study comparing cities and reporting the RR to be 1.06 per 10 µg/m^3^ increment in the annual average PM2.5 [100]. However, there is now within the research community a focus on the different types of particles and a reasoning that it is likely that their impacts on mortality differ [97]. ExternE [81] for example makes different assumptions about the toxicity of different types of PM.

One study used the ACS subjects from Los Angeles County [101]. The authors used kriging and data from 23 monitoring stations and assigned exposure estimates to 267 areas in Los Angeles with a total of 22,905 subjects. For all-cause mortality, the RR for PM2.5 was 1.17 (95% CI 1.05–1.30) per 10 μg/m^3^. These results suggest that the chronic health effects associated with PM2.5 from local sources, mainly traffic and heating, are much larger than reported when the effects of background levels in metropolitan areas are used. The direct comparison with the ACS results show effects that are nearly three times larger than in models relying on intercommunity exposure contrasts.

A recent review collected information on studies of mortality and long-term exposure to the combustion-related particle indicators [99]. The included studies used different methods, and their relation and conversion factors have been described previously [102]. All-cause mortality was significantly associated with elemental carbon, and the meta-analysis resulted in an RR of 1.061 per 1 μg/m^3^ EC (95% CI 1.049–1.073). The conversion from PM exhaust to EC is complicated due to a lack of data and varying fuel and vehicle fleet characteristics. The RR for EC (1.061 per 1 μg/m^3^) means that an assumption that EC forms one third of the urban PM results in an RR for PM2.5 that is close to the coefficient reported for Los Angeles.

Several epidemiological studies with a fine spatial resolution that can capture the gradients in exposure to local traffic pollutants did not use NOx or NO_2_ as exhaust indicators. One study from Oslo included 16,000 men, of whom 25% died during the follow up [103]. This cohort, with people 40–49 years of age at the start of the study, was followed from 1972/73 through 1998. When the median concentration of NOx in 1974–78 was used, the RR for total nonviolent mortality was 1.08 per 10 µg/m^3^ (95% CI 1.06–1.11%).

Despite the fact that these impact assessments have focused on changes in motor vehicle traffic and thus traffic air pollution, most studies have used an exposure-response function dominated by between-city comparisons [99,100], likely reflecting the effect of differences in the regional background of PM2.5. Only two studies used exposure-response functions that can be considered more representative for combustion-related particles [37,40].

### Health impacts

In most studies, the change in transport mode from motorized to active transport resulted in small air pollution-related health benefits for the general population and slightly increased risks from air pollution for active commuters. Only three studies, Grabow et al. [50], Dhondt et al. [40], and Johansson et al. [37], calculated a large benefit for the general population. Two possible reasons for the large benefits are large assumed decreases in motorized transport and the use of different particle measures, e.g. EC used by Dhondt et al. [40] and BC used by Johansson et al. [37], or the inclusion of O_3_ as in Grabow et al. [50].

The use of exposure-response functions from studies comparing background particle concentration between cities to estimate the health impacts of changes in local traffic emissions can be questioned with respect to the relevance. Background PM2.5 is often dominated by secondary particles such as sulfates and nitrates with other size and toxicity than emitted exhaust particles such as diesel soot [99,104].

Some studies used HEAT estimates based on an RR from Andersen et al. [105] to calculate the physical activity impact on mortality. The risk estimate from Andersen et al. [105] was not adjusted for air pollution exposure, and this might have reduced the beneficial effect of physical activity on all-cause mortality. Some studies, such as Rabl et al. [49] and Rojas-Reuda et al. [38,44], used the physical activity RR from Andersen et al. [105] and also considered the impact of air pollution among active commuters, which could result in a double counting of air pollution effects and an underestimation of the decrease in mortality risk related to active travel among commuters. In other words, one can assume that the negative impact of air pollution among active commuters is likely to be overestimated in these studies. However, to a lesser extent this is not likely if the air pollution level in the study area of the HIA is higher than in the epidemiological study area (Copenhagen) of Andersen et al. [32].

## Conclusion

In this study we have critically reviewed the handling of air pollution in recently published HIAs regarding a mode shift to increase active travel. Some key findings of our review are as follows.

### Type of scenarios

Almost all included studies focused on future hypothetical transport scenarios of increased cycling to give some criteria based on trip duration and distances according to which motorized trips would be shifted to cycling. Although few studies relied on individual-level survey data, most used the average estimates of cycling distances and duration for each trip due to the unavailability of reliable data. However, there are some exceptions, such as Johansson et al. [37], who employed individual-level data on people’s home and work addresses, and Buekers et al. [47], who used the actual data from reports of bicycle highway usage to construct realistic bicycle-travel scenarios. Rojas-Rueda et al. [38] and Woodcock et al. [39] evaluated public bicycle-sharing programs, assuming that 90% of new cycling trips were previously travelled by car [38] and relied on survey responses from registered users [39] in order to avoid the unrealistic assumptions described above.

### Emissions

PM 2.5 was most frequently chosen as the exposure indicator with some exceptions. Traffic emissions were calculated using model-based emission factors derived from different emission models in the majority of these studies. The only notable difference is that some studies used the proportion of concentrations generated from traffic instead of emission-based calculations.

### Population exposure and impacts

For estimation of air pollution impacts in the general population, changes in air pollution exposure due to a mode shift were estimated with the help of dispersion models in most studies. Some used a different approach by calculating benefits directly from emissions and reported the change in health cost by using published cost estimates.

### Exposure in traffic

Regarding the estimation of air pollution exposure among active commuters, the studies focused on calculating inhaled dose based on mode-specific air pollutant concentration, ventilation rate, and trip duration. The basic difference among these studies was the assumptions about mode-specific factors, including ventilation rate. Most relied on mode-specific exposure estimates taken from previous studies, whereas some either used the background urban concentration or fixed monitoring site concentration and applied mode-specific scaling factors to depict the mode-specific exposure. Almost all studies considered a concentration difference between car and bicycle and mentioned higher concentrations in car users (PM 2.5: 1.16–1.6 times or 50%, and EC: 1.65 times) than cyclists except one study where the average estimates from two street monitoring sites were used as a proxy for both car users and cyclists [42]. For the estimation of mode-specific ventilation rate, the studies either assumed a 2–8 times higher ventilation rate in cyclists than car users based on METs or 2.2 times higher rates in cyclists than car users based on average estimates from two Dutch studies [95,96].

### RR for air pollutants and health impacts

Health impacts due to changes in transport mode from car to bicycle were most often calculated using exposure-response functions between PM2.5 and all-cause mortality and diseases derived from the literature such as Krewski et al. [106], Beelen et al. [107], and Pope et al. [100,104]. Regarding the health impact of air pollution, small health benefits within the general population and small health risks for active commuters (of only a few percent) due to increased cycling were found in most studies based on exposure-response functions from studies with comparisons of exposure between cities. Such exposure-response functions seem to underestimate the impact of vehicle exhaust exposure [108]. Three studies showed larger benefits for the general population, including the same benefits as associated with physical activity (50%) [50], larger than twice the benefits as associated with physical activity [40], and twice as large as the benefits associated with the introduction of a congestion tax in central Stockholm [37].

### Knowledge gaps and future research needs

The following methodological issues and knowledge gaps that need to be tackled in future research were identified:

- Discrepancies between studies regarding the estimation of air pollution exposures and the quantification of health benefits (for both the general population and active commuters) hinder comparisons between studies.
- The use of PM2.5 as a proxy for combustion-related particles can result in underestimation of the air pollution impact on mortality due to the exclusion of other particles in vehicle exhaust. For example, BC has approximately 10 times higher relative risk than PM2.5 per mass concentration.
- Assessment of both population and commuter exposure should be included and exposure-response functions should represent the type of traffic pollution exposure that will change.
- Large variations between studies in assumed ventilation rate when cycling is affecting the estimation of air pollution effects and should be further explained.
- Data on in-traffic air pollution exposure along routes are limited, although most studies used mode-specific estimates from the literature. Further, studies relying on fixed monitoring stations to estimate in-traffic air pollution can cause underestimation of air pollution exposure for commuters.
- Estimation of health benefits for adults due to decreases in air pollution can lead to faulty conclusions because only a few studies have estimated the health impacts on children.
- Air pollution impact estimates for morbidity outcomes often only include short-term effects of air pollution, such as hospital admissions, which do not reflect the long-term effects on disease incidence.
- The research focus is now on developed countries, but information regarding developing countries such as India and China where an increase in motorized transport has become a significant source of air pollution due to a sharp increase in the number of vehicles needs further exploration.
- A more general aspect is that the use of hypothetical scenarios lacking information of actual trips, travel distances, traffic flows, and the physical capacity to change mode of transport to cycling makes comparisons between studies difficult.

## Paper context

HIA studies of increased cycling reported beneficial effects on health and environment, although with varying validity and precision. Our review has identified a large discrepancy between studies regarding air pollution exposure assessment. The use of PM2.5 as indicator and exposure response functions from between cities comparison may underestimate the impact of changes in exposure. These findings may guide future HIAs.
